# Association of plasma biomarkers with cognition, cognitive decline, and daily function across and within neurodegenerative diseases: Results from the Ontario Neurodegenerative Disease Research Initiative

**DOI:** 10.1002/alz.13560

**Published:** 2023-12-17

**Authors:** Erlan Sanchez, Tim Wilkinson, Gillian Coughlan, Saira Mirza, Andrée‐Ann Baril, Joel Ramirez, Malcolm A. Binns, Sandra E. Black, Michael Borrie, Allison A. Dilliott, Roger A. Dixon, Dar Dowlatshahi, Sali Farhan, Elizabeth Finger, Corinne E. Fischer, Andrew Frank, Morris Freedman, Rafaella A. Goncalves, David A. Grimes, Ayman Hassan, Robert A. Hegele, Sanjeev Kumar, Anthony E. Lang, Connie Marras, Paula M. McLaughlin, Joseph B. Orange, Stephen H. Pasternak, Bruce G. Pollock, Tarek K. Rajji, Angela C. Roberts, John F. Robinson, Ekaterina Rogaeva, Demetrios J. Sahlas, Gustavo Saposnik, Michael J. Strong, Richard H. Swartz, David F. Tang‐Wai, Maria Carmela Tartaglia, Angela K. Troyer, Hlin Kvartsberg, Henrik Zetterberg, Douglas P. Munoz, the ONDRI Investigators, Mario Masellis

**Affiliations:** ^1^ L.C. Campbell Cognitive Neurology Research Unit, Hurvitz Brain Sciences Program Sunnybrook Research Institute Toronto Ontario Canada; ^2^ Department of Neurology Massachusetts General Hospital, Harvard Medical School Boston Massachusetts USA; ^3^ Division of Neurology Department of Medicine University of Toronto Toronto Ontario Canada; ^4^ Douglas Mental Health University Institute McGill University Montreal Quebec Canada; ^5^ Dr. Sandra Black Centre for Brain Resilience & Recovery Hurvitz Brain Sciences Research Program Sunnybrook Research Institute Toronto Ontario Canada; ^6^ Rotman Research Institute Baycrest Health Sciences Toronto Ontario Canada; ^7^ Dalla Lana School of Public Health University of Toronto Toronto Ontario Canada; ^8^ Lawson Health Research Institute London Ontario Canada; ^9^ Schulich School of Medicine & Dentistry Western University London Ontario Canada; ^10^ Department of Neurology and Neurosurgery Montreal Neurological Institute‐Hospital McGill University Montreal Quebec Canada; ^11^ Department of Psychology Neuroscience and Mental Health Institute University of Alberta Edmonton Alberta Canada; ^12^ Department of Medicine University of Ottawa Ottawa Ontario Canada; ^13^ Ottawa Hospital Research Institute Ottawa Ontario Canada; ^14^ Keenan Research Centre for Biomedical Research Li Ka Shing Knowledge Institute St. Michael's Hospital Toronto Ontario Canada; ^15^ Bruyere Research Institute University of Ottawa Ottawa Ontario Canada; ^16^ Department of Medicine Mt. Sinai Hospital Toronto Ontario Canada; ^17^ Centre for Neuroscience Studies Queen's University Kingston Ontario Canada; ^18^ Brain and Mind Research Institute Ottawa Ontario Canada; ^19^ Thunder Bay Regional Research Institute Northern Ontario School of Medicine Thunder Bay Ontario Canada; ^20^ Robarts Research Institute Western University London Ontario Canada; ^21^ Centre for Addiction and Mental Health Toronto Ontario Canada; ^22^ Department of Psychiatry University of Toronto Toronto Ontario Canada; ^23^ Edmond J. Safra Program in Parkinson's Disease and the Morton and Gloria Shulman Movement Disorders Centre Toronto Western Hospital Toronto Ontario Canada; ^24^ Nova Scotia Health Halifax Nova Scotia Canada; ^25^ Department of Medicine Dalhousie University Halifax Nova Scotia Canada; ^26^ School of Communication Sciences and Disorders Western University London Ontario Canada; ^27^ Canadian Centre for Activity and Aging Western University London Ontario Canada; ^28^ Department of Clinical Neurological Sciences Robarts Research Institute Western University London Ontario Canada; ^29^ Parkwood Institute, St. Joseph's Health Care Centre Western University London Ontario Canada; ^30^ Department of Psychiatry University of Toronto Toronto Ontario Canada; ^31^ Department of Pharmacology and Toxicology University of Toronto Toronto Ontario Canada; ^32^ Tanz Centre for Research in Neurodegenerative Diseases University of Toronto Toronto Ontario Canada; ^33^ McMaster University Hamilton Ontario Canada; ^34^ Stroke Outcomes and Decision Neuroscience Unit, Li Ka Shing Knowledge Institute St. Michael's Hospital Toronto Ontario Canada; ^35^ Canadian Institutes for Health Research Ottawa Ontario Canada; ^36^ Sunnybrook Research Institute Sunnybrook Health Sciences Centre Toronto Ontario Canada; ^37^ Krembil Brain Institute Memory Clinic University Health Network Toronto Ontario Canada; ^38^ Department of Psychology University of Toronto Toronto Ontario Canada; ^39^ Neuropsychology and Cognitive Health Baycrest Health Sciences Toronto Ontario Canada; ^40^ Department of Psychiatry and Neurochemistry Institute of Neuroscience and Physiology The Sahlgrenska Academy University of Gothenburg Gothenburg Sweden; ^41^ Clinical Neurochemistry Laboratory Sahlgrenska University Hospital Mölndal Sweden; ^42^ Dementia Research Institute Department of Neurodegenerative Disease UCL Institute of Neurology Queen Square London UK; ^43^ Hong Kong Center for Neurodegenerative Diseases Hong Kong China; ^44^ Wisconsin Alzheimer's Disease Research Center University of Wisconsin Madison Wisconsin USA

**Keywords:** activities of daily living, amyloid, amyloid beta, blood, blood‐based, cognition, dementia, glial fibrillary acidic protein, longitudinal, neurofilament light chain, neuropsychiatric, phosphorylated tau, protein, tau, vascular

## Abstract

**INTRODUCTION:**

We investigated whether novel plasma biomarkers are associated with cognition, cognitive decline, and functional independence in activities of daily living across and within neurodegenerative diseases.

**METHODS:**

Glial fibrillary acidic protein (GFAP), neurofilament light chain (NfL), phosphorylated tau (p‐tau)181 and amyloid beta (Aβ)_42/40_ were measured using ultra‐sensitive Simoa immunoassays in 44 healthy controls and 480 participants diagnosed with Alzheimer's disease/mild cognitive impairment (AD/MCI), Parkinson's disease (PD), frontotemporal dementia (FTD) spectrum disorders, or cerebrovascular disease (CVD).

**RESULTS:**

GFAP, NfL, and/or p‐tau181 were elevated among all diseases compared to controls, and were broadly associated with worse baseline cognitive performance, greater cognitive decline, and/or lower functional independence. While GFAP, NfL, and p‐tau181 were highly predictive across diseases, p‐tau181 was more specific to the AD/MCI cohort. Sparse associations were found in the FTD and CVD cohorts and for Aβ_42/40_.

**DISCUSSION:**

GFAP, NfL, and p‐tau181 are valuable predictors of cognition and function across common neurodegenerative diseases, and may be useful in specialized clinics and clinical trials.

## 1 BACKGROUND

Cognitive impairment, dementia, and motor dysfunction are increasingly prevalent with pathological forms of aging causing an enormous burden on individuals and society.^1^ Previously identified relevant and accurate biomarkers with the ability to help diagnose and to monitor dementia (e.g., cerebrospinal fluid [CSF] tau and amyloid beta [Aβ] levels, Aβ positron emission tomography [PET])^2^ are expensive, not widely available or easily accessible, and therefore do not have great potential for scalability in the general population. Recent advances in ultra‐sensitive blood‐based immunoassays now offer accessible and cost‐effective quantification of emerging plasma biomarkers with high potential to translate to clinical settings and revolutionize how neurodegenerative diseases are diagnosed and monitored.^3^ These include glial fibrillary acidic protein (GFAP; a glial neuroinflammatory biomarker), neurofilament light chain (NfL; a neuroaxonal damage biomarker), phosphorylated tau181 (p‐tau181; a phospho‐tau isoform considered specific to Alzheimer's disease [AD] pathology), and the ratio of Aβ 42 to Aβ 40 (Aβ_42/40_; a marker of amyloid plaque deposition also considered specific to AD pathology; see Hansson et al.^4^ for an overview). A growing body of evidence has shown that plasma biomarkers—particularly p‐tau181—can effectively discriminate some disease stages and different neurodegenerative diseases.^5^, ^6^, ^7^, ^8^, ^9^, ^10^, ^11^

However, more research is needed to determine the extent to which these biomarkers relate to cross‐sectional cognitive function, longitudinal cognitive decline, and functional independence in clinically diagnosed patients with neurodegenerative diseases associated with dementia and/or motor dysfunction. This is imperative to understand better the value of plasma biomarkers to predict disease severity before widespread implementation as prognostic tools in specialized clinics and as screening and monitoring tools in clinical trials. Furthermore, most initiatives have until now focused on a single disease spectrum, primarily AD, but mixed overlapping pathologies are the rule in late‐onset sporadic dementia rather than the exception.^12^, ^13^, ^14^, ^15^ It is therefore critical that these biomarkers be characterized and related to baseline and longitudinal changes in cognition not only in AD, but also within other commonly contributing neurodegenerative diseases, such as Parkinson's disease (PD), frontotemporal dementia (FTD) spectrum disorders, and cerebrovascular disease (CVD), and across all these diseases as a whole.

We aimed here to investigate how plasma GFAP, NfL, p‐tau181, and Aβ_42/40_ are associated with (1) performance on five cognitive domains at baseline, (2) cognitive decline on five cognitive domains over a 2‐year follow‐up period, and (3) functional independence in activities of daily living (ADL) at baseline, all across and within multiple neurodegenerative and cerebrovascular diseases. Considering what these plasma biomarkers are measuring, we expected outcomes to be generally associated with GFAP and NfL levels across all diseases, but to be mostly associated with p‐tau181 in an AD‐specific manner. For these aims, we leveraged the Ontario Neurodegenerative Disease Research Initiative (ONDRI), a unique cohort with a wide breadth of longitudinal data and extensive harmonized protocols across sites and diseases.^16^, ^17^

## 2 METHODS

### 2.1 Ontario Neurodegenerative Disease Research Initiative cohort

The characteristics and processes of the ONDRI cohort have been described previously with detailed inclusion and exclusion criteria.^16^, ^17^ All participants included in this study (*n* = 480) from the ONDRI cohort had previously been diagnosed with AD or mild cognitive impairment due to AD (AD/MCI; *n* = 126), PD (*n* = 140), FTD (*n* = 53), or CVD (*n* = 161). The ONDRI amyotrophic lateral sclerosis (ALS) participants were not included in this study due to lack of cognitive impairment and follow‐up visits occurring at shorter intervals from the rest of the cohorts due to the more rapid decline observed. Participants were recruited for the ONDRI study through clinicians at tertiary clinics, between July 2014 and March 2017, at 14 academic health science centers in six cities across Ontario, Canada. In total, 584 (excluding ALS) participants were recruited; some did not meet inclusion criteria after screening (*n* = 91, of which *n* = 9 were transferred to another diagnostic group), and a few withdrew consent (*n* = 22), leaving a total of 480 participants enrolled in the study. All participants were screened on general and disease‐specific inclusion and exclusion criteria by academically practicing board‐certified neurologists, geriatric psychiatrists, or geriatricians depending on clinical diagnostic group. A board‐certified neuroradiologist reviewed the magnetic resonance imaging (MRI) scans to exclude any incidental findings and to assist with identification of supportive imaging features (e.g., for specific diagnostic groups such as stroke and AD/MCI). MRI, blood work (e.g., to exclude contributing factors for cognitive impairment), and clinical history and exam were used to support the ONDRI diagnoses. For the CVD group, a stroke physician determined the severity of white matter hyperintensity burden based on the following checklist included on the Clinical Report Form: none, mild to moderate, or extensive. This checklist is adapted from the Fazekas/age‐related white matter changes rating scales.^18^, ^19^ All participants met prevailing consensus diagnostic criteria for their respective disease, based on established clinical definitions at the time of enrollment.^20^, ^21^, ^22^, ^23^, ^24^, ^25^, ^26^, ^27^ Final inclusion into a specific diagnostic group was achieved by consensus review among physician members of that team.

Briefly, the AD/MCI group included typical and atypical AD presentations, in addition to single and multi‐domain amnestic MCI due to AD. The PD group included both cognitively intact participants and those with cognitive impairment or dementia. All patients had presented initially with the typical motor syndrome and none were diagnosed as having dementia with Lewy bodies initially. Ultimately, the composition of the PD group contained patients classified as cognitively unimpaired (34.3%), with MCI (37.1%), and with dementia (18.6%), and others who did not meet criteria for this classification, being between categories (10.0%). This classification is based on the Movement Disorder Society Task Force Level II guidelines, using objective cognitive impairment on neuropsychological evaluation interpreted by a board‐certified neuropsychologist, subjective cognitive decline reported by the participant or study partner, and functional impairment in instrumental ADL (iADL) scale.^28^ The FTD group was composed of various sporadic FTD spectrum diagnoses: behavioral variant FTD (40.7%), progressive supranuclear palsy (29.6%), progressive non‐fluent aphasia (14.8%), semantic dementia (9.3%), and corticobasal syndrome (5.6%). The CVD group included participants who had experienced a mild to moderate ischemic stroke event documented on MRI or computed tomography ≥ 3 months before enrolment and confirmed by radiologist, with or without cognitive impairment but with minimum Montreal Cognitive Assessment (MoCA) cut‐off score of 18, and with any level of small vessel disease burden. Participants with history of cognitive impairment or dementia before the vascular event or with large cortical strokes were excluded.

**RESEARCH IN CONTEXT**

1. **Systematic review**: We reviewed studies reporting on plasma biomarkers and cognition in Alzheimer's disease (AD) and other neurodegenerative or cerebrovascular diseases. Studies are mostly limited to a single disease spectrum and in the extent of cognitive assessments completed. Large gaps in the literature exist regarding how plasma biomarkers relate to cognitive performance, cognitive decline, and functional independence.
2. **Interpretation**: We present a comprehensive assessment of plasma biomarkers and their association with performance in five cognitive domains, longitudinal cognitive decline, and functional independence in activities of daily living across AD, Parkinson's disease, frontotemporal dementia spectrum disorders, and cerebrovascular disease. Selected plasma biomarkers show value in predicting cognitive and functional status.
3. **Future directions**: This report is an important step for future studies investigating how these plasma biomarkers can be concretely implemented as prognostic tools in specialized clinics and as screening or monitoring tools in clinical trials.

All participants underwent comprehensive clinical and neuropsychological assessments at baseline and annually, in addition to genetic and plasma biomarker assessments at baseline. All participants were required to have a study partner who knew them well, interacted with them regularly, and were able to provide collateral information about their function (in most cases, spouses). A healthy control (HC) group (*n* = 44), studied using the same ONDRI protocols in addition to brain Aβ PET as part of the Brain‐Eye Amyloid Memory (BEAM) study,^29^ was also included to be used as a reference for baseline plasma biomarker levels only and not for further analysis. The HC group consisted of cognitively normal individuals who were all amyloid negative on florbetapir PET. Assessment‐specific and ONDRI‐wide quality control procedures to ensure accurate data are described elsewhere.^30^, ^31^

### 2.2 Plasma and genetic biomarkers

Blood samples were drawn from all participants at baseline at the closest LifeLabs Medical Laboratory Services location to their residence, with standardized operating protocols for collection and storage (https://www.lifelabs.com/page‐section/specimen‐collection‐handling‐section‐hcp‐requisitions‐page/). Samples were collected and processed within 24 hours; they were shipped on ice pack (not frozen prior to sample processing) overnight to the OBI Biobank Sample Reception at the Robarts Research Institute (Western University, London, ON, Canada) where they were immediately processed upon receipt for plasma isolation by centrifugation at 2000 rpm for 15 minutes at 4°C, and then stored at −80°C until shipment to the Clinical Neurochemistry Laboratory (University of Gothenburg, Mölndal, Sweden) for measurement. The concentrations of GFAP, NfL, Aβ_42_, and Aβ_40_ were measured using the Neurology 4‐plex E ultra‐sensitive Single molecule array (Simoa) immunoassays, while p‐tau181 was measured using the pTau‐181 Advantage kit. The measurements were performed on an HD‐X Analyzer according to instructions from the manufacturer (Quanterix). The measurements were performed in one round of experiments using one batch of reagents by board‐certified laboratory technicians who were blinded to clinical data. Intra‐assay coefficients of variation were below 10% for all analytes. For analyte concentrations below the functional lower limit of quantification (GFAP = 11.6 pg/mL, NfL = 1.6 pg/mL, p‐tau181 = 2 pg/mL, Aβ_42_ = 1.51 pg/mL, Aβ_40_ = 4.08 pg/mL), missing data were imputed with the lower limit divided by two. Across all participants, including HC, there were 17 samples (3.2%) with concentrations below the limit for p‐tau181 (4 HC, 3 AD/MCI, 4 PD, 1 FTD, 5 CVD), 25 samples (4.8%) for Aβ_42_ (7 AD/MCI, 6 PD, 4 FTD, 8 CVD), and 7 samples (1.3%) for Aβ_40_ (3 AD/MCI, 2 PD, 2 FTD). Aβ_42/40_ ratio was not calculated for samples with Aβ_42_ or Aβ_40_ concentrations below the limit. As a general index of directionality, higher GFAP, NfL, and p‐tau181 are considered more pathological, while lower Aβ_42/40_ is considered more pathological. Apolipoprotein E (*APOE*) genotypes for each participant were identified using the custom next‐generation sequencing‐based panel ONDRISeq^32^, ^33^ and used to determine *APOE* ε4 carrier status. Less than 3% of the ONDRI cohort had monogenic mutations in genes known to be drivers of neurodegenerative diseases.^34^

### 2.3 Cognitive domains

All participants underwent the ONDRI neuropsychology protocol,^30^ which provides a comprehensive assessment of cognition and behavior, at baseline and at two yearly follow‐up visits. Screening was done for English comprehension, visual acuity, and auditory acuity, which may confound test performance. Performance on five cognitive domains (attention and working memory, executive function, language, memory, and visuospatial function) were evaluated with domain‐specific composite scores adjusted for age, sex, and education. Detailed methods and the full list of neuropsychological tests by cognitive domain are presented in supporting information Section 1 and Tables S1 and S2. The composite scores were derived from 26 raw test scores from the comprehensive assessment, based on conventions in clinical neuropsychology^35^ and consensus agreement among the ONDRI neuropsychologists.

### 2.4 Independence in activities of daily living

Lawton–Brody scales completed by study partners at baseline were used to measure the participant's ability to function independently across activities of daily living.^36^ The Physical Self Maintenance (bADL) scale includes feeding, dressing, grooming, ambulation, bathing, and toileting. The iADL scale includes telephone use, shopping, food preparation, housekeeping, laundering, use of transportation, managing medications, and financial management. Percentage scores reflecting functional independence, for which higher scores reflect greater independence, were computed for both scales. Study partners had the option to rate iADL items as not applicable, which reduced the maximum obtainable score used in iADL percentage scores calculations.

### 2.5 Statistical analyses

All plasma biomarkers (GFAP, NfL, p‐tau181, Aβ_42/40_) were log_10_‐transformed before analysis due to skewness. Chi‐square tests or one‐way analyses of variance with Tukey post hoc were used to compare descriptive variables (demographic, clinical, plasma biomarker) among all available groups. Independent *t* tests were used to explore sex differences in plasma biomarkers and outcome variables. As sensitivity analysis to explore the effect of attrition in the cohort, demographic, clinical, plasma biomarker, and cognitive variables were compared using independent *t* tests or chi‐square tests between those without follow‐up and those with at least one follow‐up visit.

Linear mixed models were used to assess the association among baseline plasma biomarkers, baseline age/sex/education‐adjusted cognitive domains, and longitudinal change in age/sex/education‐adjusted cognitive domains. Covariates included *APOE* ε4 carrier status (presence or absence), time, and time x plasma biomarker interaction. Participant ID was entered as a random effect. Time was defined as a continuous variable of months from initial visit at moment of testing, for all three visits included. Plasma biomarker (log) was entered as a continuous variable. Estimates for biomarker associations with baseline cognitive domain composite scores and with longitudinal change in cognitive domain composite scores were derived from this single linear mixed model. Extreme cognitive outliers (greater than −4 standard deviations) were detected for each of language and visuospatial functions (AD/MCI *n* = 1, FTD *n* = 2, CVD *n* = 1) which, while being real values, could affect statistical results. As sensitivity analyses, the models were repeated with these outliers removed. To assess the association between baseline plasma biomarkers and level of independence in activities of daily living at baseline, linear regressions were performed while controlling for age, sex, education, and *APOE* ε4 carrier status. Associative analyses were first performed in a pooled disease group of all participants to investigate these associations regardless of given diagnosis, and then with stratification to characterize these associations in individual cohorts. Secondary analyses performed in the HC cohort, and also with Aβ_42_, are present in supporting information Sections 2 and 3. Statistical analyses were performed with SPSS Statistics 29 (IBM Corp., 2022). Beta and *P*‐value SPSS outputs (B, linear mixed models; standardized β, linear regressions) are reported. The threshold of statistical significance was set at *P* < 0.05 (two tailed).

## 3 RESULTS

### 3.1 Sample characteristics

**FIGURE 1 alz13560-fig-0001:**
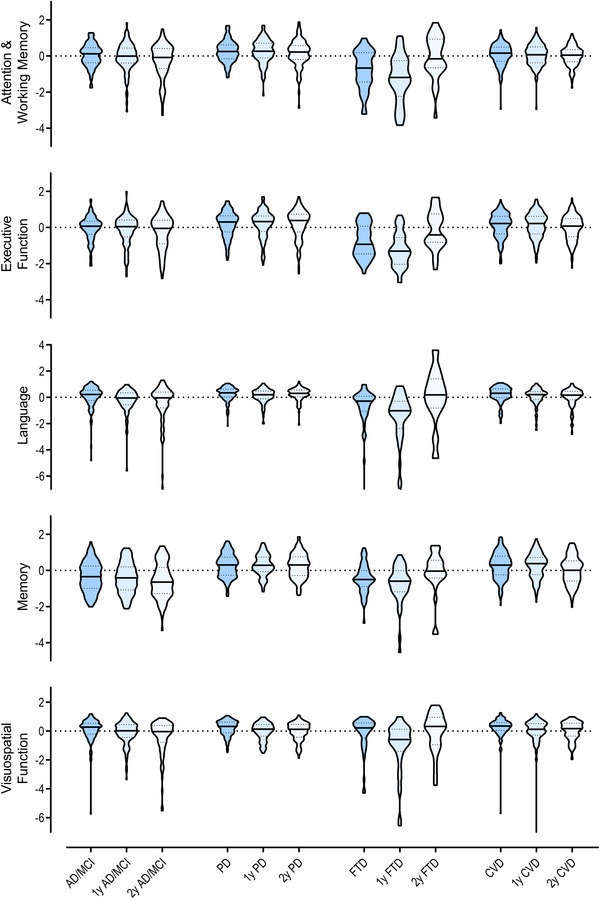
Cognitive domains at baseline and follow‐up visits across diseases. Composite *z* scores are relative to the mean and standard deviation of pooled disease groups at baseline, with the observed effects of age, sex, and education removed. Truncated violin plots do not extend past maximal values. Language and visuospatial function graphs’ y axes are cut at −7 for display purposes, excluding only a few participants for language (FTD, *n* = 1; 1y‐FTD, n = 1; 2y‐AD/MCI, *n* = 1) and visuospatial function (1y‐CVD, *n* = 1). 1y, 1‐year; 2y, 2‐year; AD/MCI, Alzheimer's disease/mild cognitive impairment; CVD, cerebrovascular disease; FTD, frontotemporal dementia; PD, Parkinson's disease.

The sample characteristics for the different ONDRI disease groups are described in Table 1, and cognitive domains at baseline and follow‐up visits are presented in Figure 1. Although all groups were similar in mean age, the FTD group had a slightly lower age range at baseline (49 to 80 years old) compared to the AD/MCI (53 to 87 years old), PD (55 to 85 years old), and CVD (54 to 85 years old) groups, which is consistent with FTD spectrum disorders. Of note, the FTD group also had fewer years[Fig alz13560-fig-0001] of education, was considerably worse in most clinical measures, and had the most attrition of all groups. Overall, the cohort consisted of 67.1% men, with a lower proportion in the AD group (54.8%). Women had slightly higher GFAP levels than men across the cohort (*t* = 5.34, *P* < 0.001), with no differences for other plasma biomarkers. There were also no differences between men and women in cognitive function or independence in ADLs. The HC group had a slightly lower age range (51 to 77 years old) and a much lower proportion of men (24.4%) than the disease cohorts.

**TABLE 1:** Baseline sample demographic, clinical, biomarker, and genetic characteristics.

	All diseases	AD/MCI	PD	FTD	CVD	HC	*P* value	Effect sizes
N at baseline	480	126	140	53	161	44	–	–
N at 1‐year follow‐up	429	108	125	43	153	–	–	–
N at 2‐year follow‐up	367	94	106	30	137	–	–	–
Age, years	69.2 (7.4)	71.0 (8.2)	67.9 (6.3)	67.8 (7.1)	69.2 (7.4)	66.7 (6.0)	*P* < 0.001^a^	*η* ^2^ = 0.035
Sex, % of men	67.1%	54.8%	77.9%	64.2%	68.3%	24.4%	*P* < 0.001^b^	w = 0.302
*APOE* ε4 carriers, %	32.3%	49.2%	23.6%	37.7%	24.8%	22.7%	*P* < 0.001^c^	w = 0.231
Education, years	15.0 (2.9)	15.2 (3.1)	15.5 (2.7)	13.9 (2.7)	14.6 (2.9)	16.3 (2.0)	*P* < 0.001^d^	*η* ^2^ = 0.047
MoCA, total score	24.3 (3.4)	22.7 (3.0)	25.8 (2.6)	21.5 (3.9)	25.2 (3.0)	28.1 (1.4)	*P* < 0.001^e^	*η* ^2^ = 0.286
NPI, total severity	4.0 (4.5)	3.7 (4.0)	3.5 (3.9)	8.2 (6.2)	3.1 (3.9)	–	*P* < 0.001^f^	*η* ^2^ = 0.073
mRS, score	–	–	–	–	1.0 (0.8)	–	–	–
MDS‐UPDRS, total score	–	–	46.5 (19.7)	–	–	–	–	–
MDS‐UPDRS Part III, score	–	–	23.2 (11.8)	–	–	–	–	–
bADL, % of independence	96.5 (8.3)	98.2 (4.6)	96.6 (7.3)	87.8 (15.6)	98.1 (5.5)	–	*P* < 0.001^g^	*η* ^2^ = 0.148
iADL, % of independence	85.7 (19.3)	85.3 (17.3)	89.7 (14.1)	61.5 (27.6)	90.8 (14.7)	–	*P* < 0.001^h^	*η* ^2^ = 0.217
GFAP, pg/mL	109.4 (89.9)	143.1 (132.5)	88.7 (58.3)	110.4 (52.6)	100.7 (72.4)	91.5 (34.3)	*P* < 0.001^i^	*η* ^2^ = 0.068
NfL, pg/mL	26.2 (21.6)	27.4 (29.8)	20.4 (10.6)	33.5 (20.5)	27.7 (20.5)	17.5 (5.6)	*P* < 0.001^j^	*η* ^2^ = 0.074
P‐tau181, pg/mL	9.9 (32.6)	12.8 (27.4)	6.7 (3.8)	8.0 (5.6)	7.1 (6.3)	4.4 (2.6)	*P* < 0.001^k^	*η* ^2^ = 0.075
Aβ_42/40_ ratio	0.064 (0.015)	0.061 (0.018)	0.066 (0.014)	0.062 (0.011)	0.065 (0.014)	0.064 (0.014)	*P* = 0.015^l^	*η* ^2^ = 0.025

*Notes*: Data are presented as mean (standard deviation) when applicable. Group comparisons are performed on log_10_‐transformed plasma biomarkers.

Abbreviations: Aβ, amyloid beta; AD/MCI, Alzheimer's disease/mild cognitive impairment; *APOE*, apolipoprotein E; bADL, basic activities of daily living; CVD, cerebrovascular disease; FTD, frontotemporal dementia; GFAP, glial fibrillary acidic protein; HC, healthy control; iADL, instrumental activities of daily living; MDS‐UPDRS, Movement Disorder Society – Unified Parkinson's Disease Rating Scale; mRS, Modified Rankin Scale; MoCA, Montreal Cognitive Assessment; NfL, neurofilament light chain; NPI, Neuropsychiatric Inventory; PD, Parkinson's disease; p‐tau, phosphorylated tau.

^a^ AD/MCI > PD, HC.

^b^ AD/MCI < PD, CVD. – HC < AD/MCI, PD, FTD, CVD.

^c^ AD/MCI > PD, CVD, HC.

^d^ FTD < AD/MCI, PD, HC – CVD < HC.

^e^ AD/MCI, FTD < PD, CVD – HC > AD/MCI, PD, FTD, CVD.

^f^ FTD > AD/MCI, PD, CVD.

^g^ FTD < AD/MCI, PD, CVD.

^h^ FTD < AD/MCI, PD, CV. – AD/MCI < CVD.

^i^ AD/MCI > PD, CVD, HC – FTD > PD.

^j^ FTD > AD/MCI – AD/MCI, FTD, CVD > PD – AD/MCI, FTD, CVD > HC.

^k^ AD/MCI > PD, CVD. – AD/MCI, PD, FTD, CVD > HC.

^l^ AD/MCI < PD, CVD.

Overall, in reference to the HC group, the AD/MCI group had elevated mean levels of GFAP; the AD/MCI, FTD, and CVD groups had elevated mean levels of NfL; and the AD/MCI, PD, FTD, and CVD groups had elevated mean levels of p‐tau181. No groups were different from HC for levels of Aβ_42/40_. Differences in plasma biomarker levels between all disease groups are displayed in Table 1. Intercorrelations between plasma biomarkers levels are presented in supporting information Section 4 and Table S4.

Total attrition in the entire sample was 10.6% at second visit, and 23.5% at third visit (Table 1 includes attrition breakdown by disease group). Relative to baseline, the average interval until second visit was 12.2 ± 0.9 months (range: 10 to 17 months), while the average interval until third visit was 24.5 ± 1.3 months (range: 22 to 32 months). Early withdrawals by participants were due to a few reasons (*n* compiled at third visit): death (*n* = 8), inability to continue due to disease progression (*n* = 26), and other (*n* = 57), in addition to participants without usable neuropsychological data (*n* = 22). “Other” reasons were the following: moved, study partner illness or death, study burden, medical comorbidity, another trial, lost to follow‐up, and withdrawn consent. While age, sex, years of education, and plasma biomarker levels were not significantly different in participants with and without follow‐ups, the latter group showed greater impairments in cognitive function: participants without any follow‐ups had significantly worse attention and working memory (*t* = −2.07, *P* = 0.044), executive function (*t* = −2.32, *P* = 0.025), and visuospatial function (*t* = −2.10, *P* = 0.036) at baseline.

### 3.2 Pooled diseases regardless of given clinical diagnosis

**FIGURE 2:**
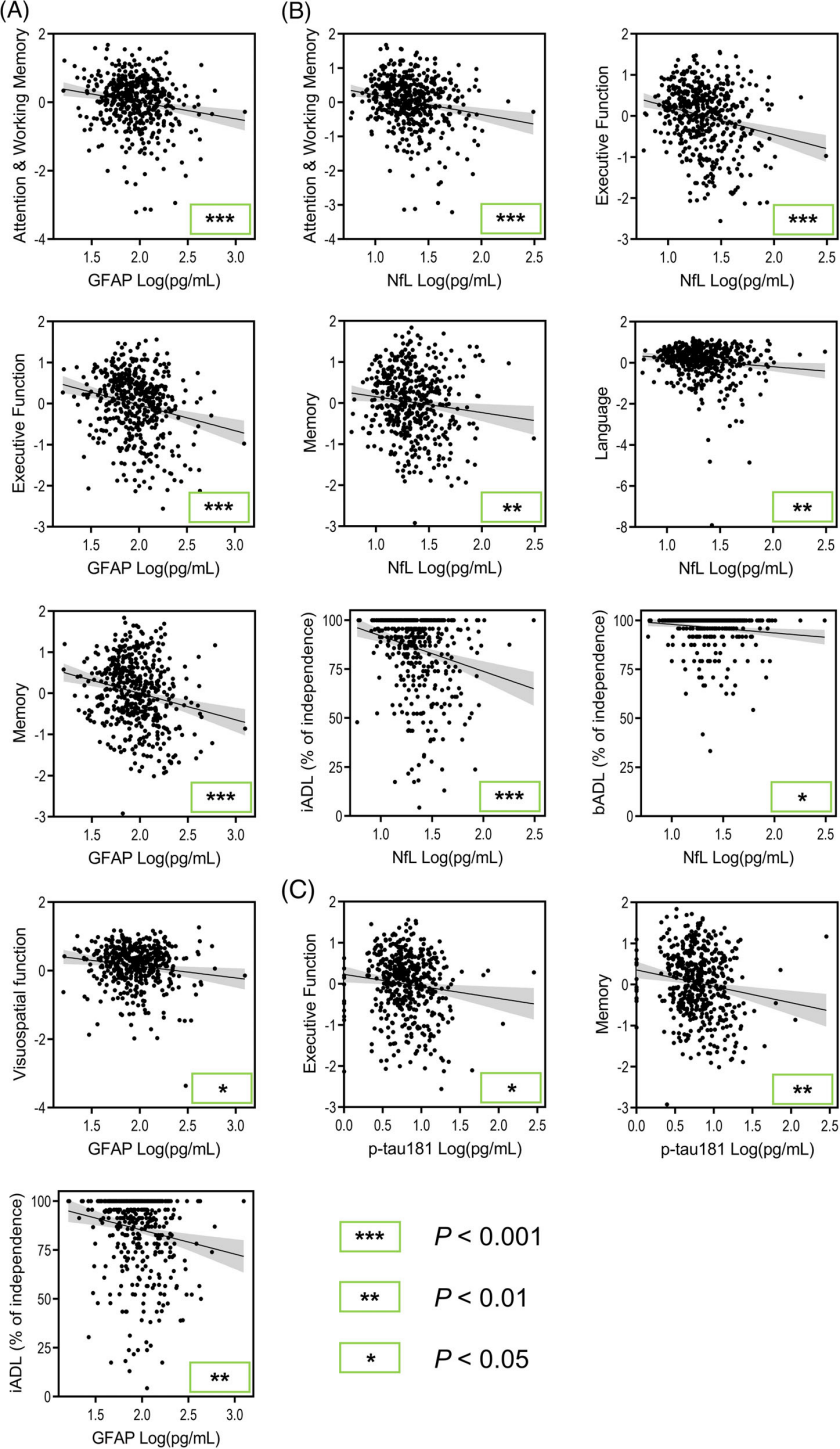
Significant plasma biomarker associations with baseline cognitive domains and independence in activities of daily living across pooled diseases regardless of given diagnosis. Age, sex, years of education, and *APOE* ε4 carrier status were accounted for in statistical models or at initial computing of cognitive domain scores. Raw data are plotted and *P* values are derived from the combined group linear mixed effect models. (A) Significant GFAP associations. (B) Significant NfL associations. (C) Significant p‐tau181 associations. The gray area represents the 95% confidence interval. *APOE*, apolipoprotein E; GFAP, glial fibrillary acidic protein; iADL, instrumental activities of daily living; NfL, neurofilament light chain; p‐tau, phosphorylated tau.

Across all neurodegenerative and cerebrovascular disease participants pooled together, GFAP, NfL, and p‐tau181 were found to be associated with most cognitive domains at baseline (Figure 2, raw data presented). Higher GFAP levels were significantly associated with worse attention and working memory (*B* = −0.52, *P* < 0.001), executive function (*B* = −0.63, *P* < 0.001), memory (*B* = −0.61, *P* < 0.001), and visuospatial function (*B* = −0.31, *P* = 0.036) scores. Higher NfL levels were significantly associated with worse attention and working memory (*B* = −0.59, *P* < 0.001), executive function (*B* = −0.67, *P* < 0.001), language (*B* = −0.49, *P* = 0.007), and memory (*B* = −0.41, *P* = 0.008) scores. Higher p‐tau181 levels were significantly associated with worse executive function (*B* = −0.26, *P* = 0.036) and memory (*B* = −0.34, *P* = 0.005) scores.

Longitudinally, higher GFAP levels were significantly associated with greater decline in attention and working memory (*B* = −0.014, *P* < 0.001), executive function (*B* = −0.013, *P* < 0.001), language (*B* = −0.018, *P* = 0.002), and visuospatial function (*B* = −0.020, *P* = 0.001) scores. Higher NfL levels were significantly associated with greater decline in attention and working memory (*B* = −0.014, *P* = 0.002), executive function (*B* = −0.011, *P* = 0.004), language (*B* = −0.023, *P* < 0.001), memory (*B* = −0.011, *P* = 0.016), and visuospatial function (*B* = −0.018, *P* = 0.010) scores. Higher p‐tau181 levels were significantly associated with greater decline in attention and working memory (*B* = −0.008, *P* = 0.016) scores. All coefficients and *P* values for baseline and longitudinal cognitive composite score associations with biomarker measures were derived from a single linear mixed effect model per cognitive domain and biomarker. Additional analyses with global cognition, and sensitivity analyses accounting for kidney and liver function, are presented in supporting information Sections 5 and 6 and Tables S5 and S6.

Regarding baseline independence in ADL (Figure 2), higher GFAP levels were significantly associated with greater impairments in iADL function (*β* = −0.16, *P* = 0.003), while higher NfL levels were significantly associated with greater impairments in both bADL (*β* = −0.12, *P* = 0.022) and iADL function (*β* = −0.21, *P* < 0.001).

### 3.3 AD/MCI

**FIGURE 3:**
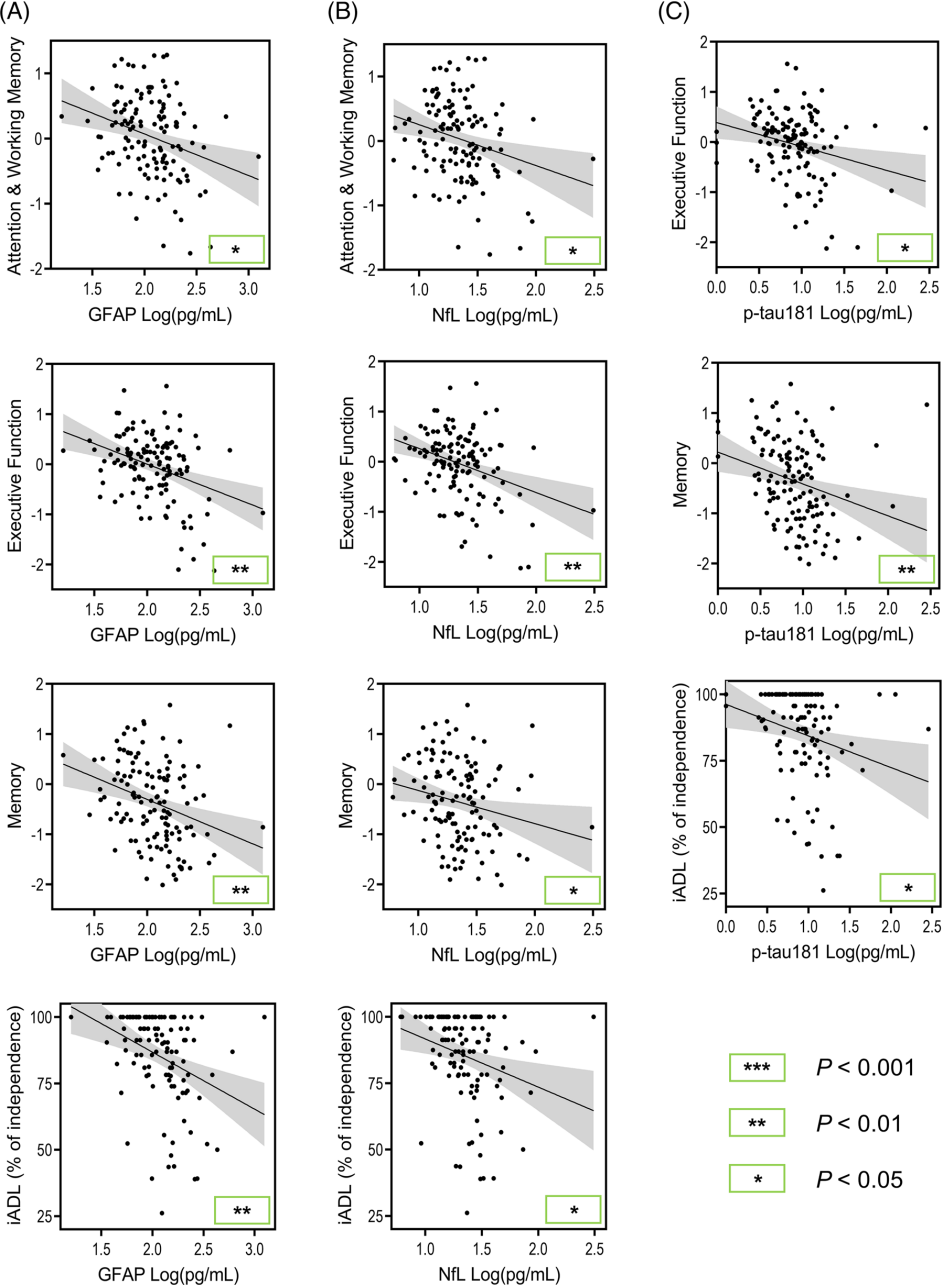
Significant plasma biomarker associations with baseline cognitive domains and independence in activities of daily living in AD/MCI. Age, sex, years of education, and *APOE* ε4 carrier status were accounted for in statistical models or at initial computing of cognitive domain scores. Raw data are plotted and *P* values are derived from the AD/MCI group linear mixed effect models. (A) Significant GFAP associations. (B) Significant NfL associations. (C) Significant p‐tau181 associations. The gray area represents the 95% confidence interval. AD/MCI, Alzheimer's disease/mild cognitive impairment; *APOE*, apolipoprotein (E) GFAP, glial fibrillary acidic protein; iADL, instrumental activities of daily living; NfL, neurofilament light chain; p‐tau, phosphorylated tau.

In the AD/MCI group, GFAP, NfL, and p‐tau181 were found to be associated with almost the same three cognitive domains at baseline (Figure 3). Higher GFAP and NfL levels were significantly associated with worse attention and working memory (*B* = −0.61, *P* = 0.011; *B* = −0.60, *P* = 0.023; respectively), executive function (*B* = −0.75, *P* = 0.002; *B* = −0.80, *P* = 0.002; respectively), and memory (*B* = −0.71, *P* = 0.005; *B* = −0.67, *P* = 0.016; respectively) scores. Higher p‐tau181 levels were significantly associated with worse executive function (*B* = −0.44, *P* = 0.025) and memory (*B* = −0.54, *P* = 0.009) scores.

Longitudinally, higher GFAP and NfL levels were significantly associated with greater decline in attention and working memory (*B* = −0.025, *P* < 0.001; *B* = −0.024, *P* = 0.003; respectively), executive function (*B* = −0.027, *P* < 0.001; *B* = −0.024, *P* = 0.002; respectively), language (*B* = −0.039, *P* < 0.001; *B* = −0.051, *P* < 0.001; respectively), and visuospatial function (*B* = −0., *P* = 0.0; *B* = −0., *P* = 0.; respectively) scores. Higher p‐tau181 levels were significantly associated with greater decline in attention and working memory (*B* = −0.012, *P* = 0.026), executive function (*B* = −0.016, *P* = 0.003), and language (*B* = −0.025, *P* = 0.004) scores. Lower Aβ_42/40_ was significantly associated with greater decline in executive function (*B* = 0.040, *P* = 0.034) scores.

Regarding baseline independence in ADL (Figure 3), higher GFAP, NfL, and p‐tau181 levels were all significantly associated with greater impairments in iADL function (*β* = −0.28, *P* = 0.004; *β* = −0.21, *P* = 0.026; *β* = −0.21, *P* = 0.020; respectively).

### 3.4 PD

**FIGURE 4:**
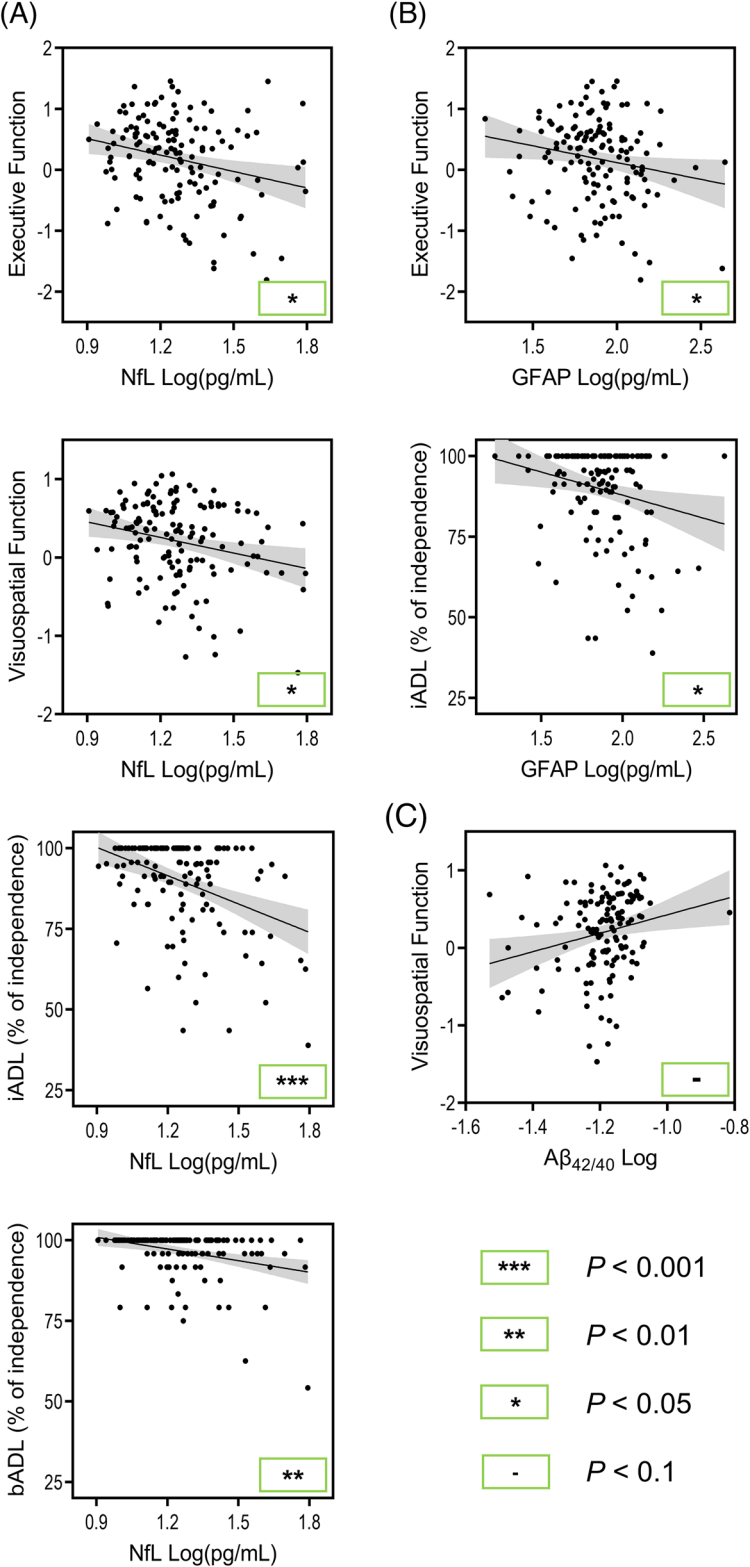
Significant plasma biomarker associations with baseline cognitive domains and independence in activities of daily living in PD. Age, sex, years of education, and *APOE* ε4 carrier status were accounted for in statistical models or at initial computing of cognitive domain scores. Raw data are plotted and *P* values are derived from the PD group linear mixed effect models. (A) Significant NfL associations. (B) Significant GFAP associations. (C) Significant Aβ_42/40_ associations. The gray area represents the 95% confidence interval. Aβ, amyloid beta; *APOE*, apolipoprotein (E) GFAP, glial fibrillary acidic protein; iADL, instrumental activities of daily living; NfL, neurofilament light chain; PD, Parkinson's disease; p‐tau, phosphorylated tau.

In the PD group, GFAP, NfL, and Aβ_42/40_ were found to be associated with many cognitive domains at baseline (Figure 4). Higher GFAP levels were significantly associated with worse executive function (*B* = −0.57, *P* = 0.039) scores. Higher NfL levels were significantly associated with worse executive function (*B* = −0.86, *P* = 0.011) and visuospatial function (*B* = −0.62, *P* = 0.012) scores. A statistically indeterminate (0.05 < *P* < 0.1) association was also present between lower Aβ_42/40_ and worse visuospatial function (*B* = 0.94, *P* = 0.057) scores.

Longitudinally, higher GFAP and NfL levels were both significantly associated with greater decline in attention and working memory (*B* = −0.016, *P* = 0.028; *B* = −0.024, *P* = 0.011; respectively) and memory (*B* = −0.025, *P* = 0.001; *B* = −0.043, *P* < 0.001; respectively) scores.

Regarding baseline independence in ADL (Figure 4), higher GFAP levels were significantly associated with greater impairments in iADL function (*β* = −0.20, *P* = 0.042), while higher NfL levels were significantly associated with greater impairments in both bADL (*β* = −0.27, *P* = 0.004) and iADL function (*β* = −0.32, *P* < 0.001).

### 3.5 FTD spectrum disorders

In the FTD group, while Aβ_42/40_ was associated with language (*B* = 6.95, *P* = 0.040) scores at baseline, this association disappeared when excluding extreme cognitive outliers (*n* = 2). Longitudinally, lower Aβ_42/40_ was significantly associated with less decline in executive function (*B* = −0.15, *P* = 0.031) scores. Regarding baseline independence in ADL, no significant associations were found.

### 3.6 CVD

**FIGURE 5:**
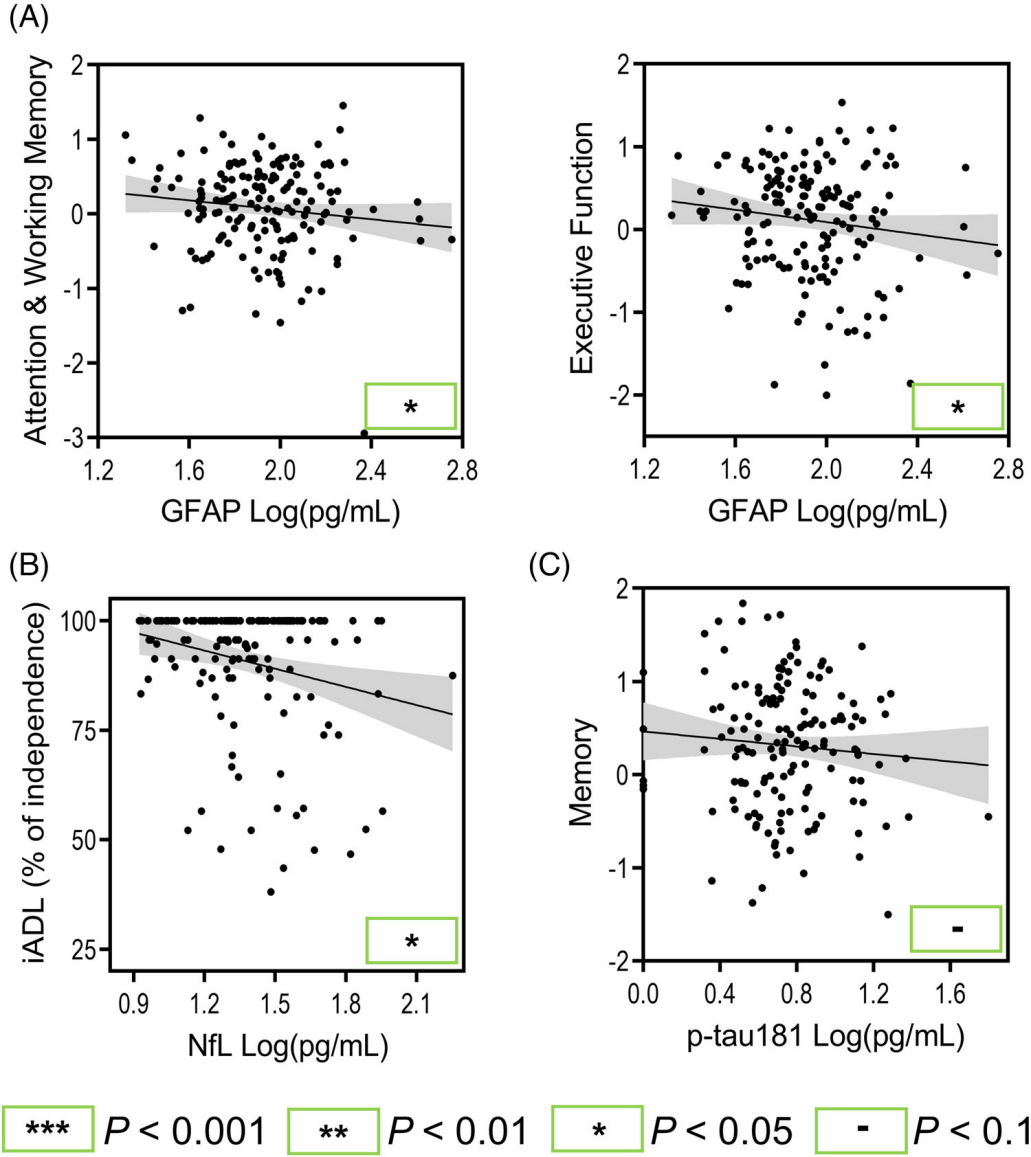
Significant plasma biomarker associations with baseline cognitive domains and independence in activities of daily living in CVD. Age, sex, years of education, and *APOE* ε4 carrier status were accounted for in statistical models or at initial computing of cognitive domain scores. Raw data are plotted and *P* values are derived from the CVD group linear mixed effect models. (A) Significant GFAP associations. (B) Significant NfL associations. (C) Significant p‐tau181 associations. The gray area represents the 95% confidence interval. *APOE*, apolipoprotein E; CVD, cardiovascular disease; GFAP, glial fibrillary acidic protein; iADL, instrumental activities of daily living; NfL, neurofilament light chain; p‐tau, phosphorylated tau.

In the CVD group, higher GFAP levels were significantly associated with worse attention and working memory (*B* = −0.42, *P* = 0.030) and executive function (*B* = −0.45, *P* = 0.044) scores at baseline (Figure 5). A statistically indeterminate (0.05 < *P* < 0.1) association was also present between higher p‐tau181 levels and worse memory (*B* = −0.32, *P* = 0.062) scores. No significant associations were found between plasma biomarkers and longitudinal change in any cognitive domains. Regarding baseline independence in ADL, higher NfL levels were significantly associated with greater impairments in iADL function (*β* = −0.22, *P* = 0.015; Figure 5).

## 4 DISCUSSION

**FIGURE 6:**
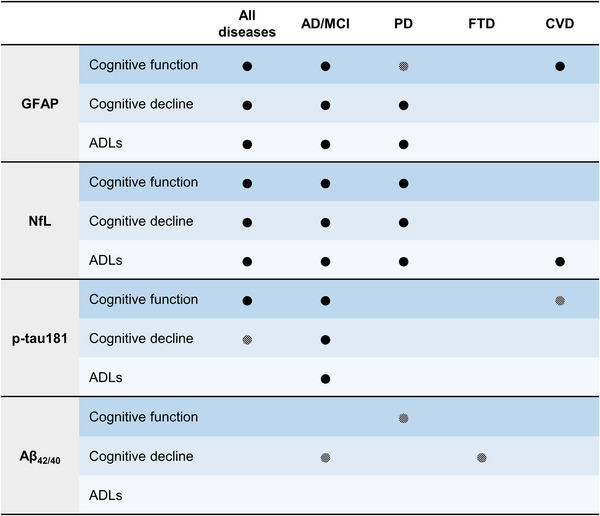
Simplified summary of the associative value of plasma biomarkers with outcome measures in the ONDRI sample. Black dot: significant associations with two or more cognitive domains, at baseline or longitudinally, or significant associations with any functional independence scale. Crosshatched dot: significant or statistically indeterminate (*P* < 0.1) association with one cognitive domain, at baseline or longitudinally. The five cognitive domains are: attention and working memory, executive function, language, memory, visuospatial function. The ADLs category includes % of independence in basic and/or instrumental ADLs. Aβ, amyloid beta; ADLs, activities of daily living; AD/MCI, Alzheimer's disease/mild cognitive impairment; CVD, cardiovascular disease; FTD, frontotemporal dementia; GFAP, glial fibrillary acidic protein; iADL, instrumental activities of daily living; NfL, neurofilament light chain; ONDRI, Ontario Neurodegenerative Disease Research Initiative; PD, Parkinson's disease; p‐tau, phosphorylated tau.

We demonstrated the association of plasma GFAP, NfL, p‐tau181, and Aβ_42/40_ with performance on several cognitive domains at baseline, domain‐specific cognitive decline over time, and functional independence in ADLs across neurodegenerative and cerebrovascular diseases in the clinical, multi‐site ONDRI cohort. A simplified summary of the associative value of these plasma biomarkers in our sample is presented in Figure 6. We also described the elevation in levels of GFAP, NfL, and/or p‐tau181 across all of these diseases compared to a healthy aging cohort. These results are key to understanding the potential utility of novel plasma biomarkers to characterize participants with diverse clinical presentations or dementia pathology for better prognosis and monitoring in specialized clinics and in clinical trials.

### 4.1 Plasma GFAP and NfL

We found higher levels of both plasma GFAP and NfL to be broadly associated with worse outcomes for most baseline cognitive domains, for cognitive decline, and for loss of functional independence in the pooled cohort of all diseases, which appeared to be driven mostly by the AD/MCI and PD cohorts in which similar associations were found. In contrast, sparse associations were found for both plasma GFAP and NfL in the CVD cohort, and none in the FTD cohort. GFAP is a marker of astrogliosis, a known pathological process of multiple neurodegenerative diseases, and has been shown to be elevated in AD/MCI, PD, and some forms of FTD.^37^, ^38^, ^39^ Similarly, NfL is a marker of neuroaxonal injury, and has been shown to be elevated in these diseases as well.^8^, ^40^, ^41^, ^42^ A few studies have shown an increase in these two plasma biomarkers along with the stage of the disease, from cognitively normal to MCI to dementia.^8^, ^9^, ^38^, ^42^, ^43^, ^44^, ^45^, ^46^ As such, our broad results are in keeping with the idea that GFAP and NfL are not specific to a single neurodegenerative disease process, and support some neuropathological overlap across these diseases, especially in AD/MCI and PD. Other studies have also investigated the association of GFAP and NfL with cognition, albeit predominantly with screening tools of global cognition (e.g., Mini‐Mental State Examination [MMSE], MoCA), and have found elevated GFAP to be associated with worse baseline cognition or cognitive decline in AD/MCI,^10^, ^47^ PD or other synucleinopathies,^38^ or FTD,^37^, ^48^ while others did not.^10^, ^49^ Similarly, studies have found elevated NfL to be associated with worse cognition or cognitive decline in AD/MCI,^8^, ^10^, ^42^, ^50^ PD or other synucleinopathies,^44^, ^45^, ^46^, ^50^, ^51^, ^52^, ^53^ or FTD,^54^ while others did not.^10^, ^47^, ^48^, ^49^, ^53^, ^55^

While the absence of findings with GFAP or NfL in the FTD cohort may be a true finding, the FTD cohort is considerably smaller than the other disease cohorts. Therefore, we would need large effect sizes for associations to be detectable in these multivariable linear models,^16^ raising the possibility that false negative findings are present, given that cognitive decline is one of the last observable changes in neurodegenerative trajectories. Furthermore, the FTD spectrum contains immense heterogeneity, both in terms of underlying predicted pathology and clinical presentations.^56^ Our FTD cohort contained a wide spectrum of predominantly sporadic subtype diagnoses, including behavioral variant FTD, progressive supranuclear palsy, progressive non‐fluent aphasia, semantic dementia, and corticobasal syndrome, which may interact with the relationship between plasma biomarkers and cognition. This lack of a finding highlights the need for large FTD cohorts that can investigate effects in the different subtypes of the FTD spectrum. For example, studies from large genetic FTD‐centered initiatives have shown that GFAP is only elevated in familial FTD due to progranulin (*GRN*) mutations,^37^ which is a TDP43 proteinopathy, and similarly, that NfL levels have different trajectories over time, with faster increases in *GRN* mutation carriers.^40^

### 4.2 Plasma p‐tau181 and Aβ_42/40_

Regarding AD‐related plasma biomarkers, we found higher levels of plasma p‐tau181 to be predominantly associated with worse outcomes for most cognitive domains, for cognitive decline, and for functional independence in the AD/MCI cohort, but also in the pooled disease group regardless of diagnosis. As p‐tau181 is a specific marker of AD brain pathology and has been shown to discriminate AD from other non‐AD neurodegenerative diseases with high accuracy,^5^, ^7^ these AD/MCI–specific results were expected. Others have shown evidence of similar associations with global cognition in the AD/MCI spectrum and of p‐tau181's predictive value for progression from MCI to dementia.^57^ Regarding levels of plasma Aβ_42/40_, overall we found very few associations with baseline cognition or cognitive decline, in keeping with the (A)myloid/(T)au/(N)eurodegeneration framework and related research showing that Aβ has finished depositing before symptomatic stages of the diseases.^58^, ^59^ Aβ_42/40_ might be a more relevant biomarker before clinical onset, as further changes are less expected in our sample of clinically diagnosed participants; a recent study demonstrated that the largest changes in plasma Aβ_42/40_ preceded brain amyloid positivity by decades.^60^ It should also be considered that immunoassays for Aβ_42/40_, including Simoa assays, have been shown to have lower performance and predictive value than mass spectrometry methods.^61^, ^62^ Still, in the PD cohort, and consistent with other studies,^49^, ^55^, ^63^ we report a notable statistically indeterminate association between lower Aβ_42/40_ ratio and worse cognition. As decreased Aβ_42/40_ ratio is a marker of early AD pathology, we can surmise that PD participants with lower Aβ_42/40_ ratio may have had early AD co‐pathology and were therefore more cognitively impaired.

While no significant associations were present between the levels of p‐tau181 and cognitive outcomes in the CVD cohort, we found a notable statistically indeterminate association between higher p‐tau181 levels and worse memory performance, which may be reflecting underlying AD co‐pathology in some of these participants. Additionally, p‐tau181 average levels in the CVD cohort were elevated compared to HCs, and for some participants were as elevated as the AD/MCI cohort, aligning with this hypothesis. This also is consistent with the often‐reported concomitant presence of AD and cerebrovascular pathology.^12^, ^14^ We also have to consider that small vessel disease as identified by T2 changes on MRI is heterogeneous, as its etiology can include any combination of cerebrovascular factors, venous collagenosis,^64^, ^65^ and cerebral amyloid angiopathy (CAA, an AD‐related vasculopathy).^66^, ^67^ Therefore, the statistically indeterminate association between elevated p‐tau181 and greater memory decline in the CVD cohort may be indicative of CVD pathology driven by CAA, which could be more harmful to cognition. Indeed, a previous study hypothesized that CAA might be the most likely etiology of small vessel disease in *APOE* ε4 carriers and may have a multiplicative detrimental effect on cognition compared to non‐carriers, supporting this hypothesis.^66^

### 4.3 Strengths and limitations

We note two overall strengths of our study. First is the ONDRI cohort's multiple well‐phenotyped disease diagnostic groups, with harmonized testing and processing protocols throughout, which allow valid comparisons among disease cohorts. Second, the assessment of cognitive domains with yearly comprehensive neuropsychological batteries and of valid functional independence assessments by study partners represent a considerable strength compared to screening measures of global cognition that lack granularity and sensitivity, but which have been used much more commonly due to ease of access and administration.

We also acknowledge a few limitations of our study. First, we did not have PET, CSF, or autopsy‐confirmed diagnoses of AD pathology, which makes it more probable that participants had unknown mixed pathology contributing to their assigned clinical diagnosis, reflecting a very common clinical reality. As elevated p‐tau181 levels were found in non‐AD neurodegenerative diseases, although at about half the rate found in AD, it is likely that some AD co‐pathology was present in these cohorts. Second, we acknowledge the lack of diversity within the ONDRI cohort, with the majority (>80%) being of European descent,^16^ which may potentially limit the generalizability of our findings to other ethnicities. Third, the unbalanced number of diagnoses might also bias the associations found in the pooled cohort analyses. Finally, although our longitudinal results are novel, they are limited by the 2‐year follow‐up timeframe, which is a relatively narrow window into the disease continuum of dementia, and by asymmetric attrition possibly introducing a positive bias in subsequent yearly cognitive assessments.

## 5 CONCLUSION

Altogether, we present a comprehensive assessment of the associations among plasma biomarkers and detailed cognitive domains, longitudinal cognitive decline, and functional independence in ADLs across multiple common neurodegenerative and cerebrovascular diseases. All plasma biomarkers showed some utility in predicting these outcomes, with GFAP and NfL being highly effective regardless of given diagnosis, and p‐tau181 being highly effective mostly in the AD/MCI population. P‐tau181 and/or Aβ_42/40_ ratio may still help identify mixed pathologies, especially in the PD or CVD populations.

## CONFLICT OF INTEREST STATEMENT

H.Z. has served on scientific advisory boards and/or as a consultant for Abbvie, Acumen, Alector, Alzinova, ALZPath, Annexon, Apellis, Artery Therapeutics, AZTherapies, CogRx, Denali, Eisai, Nervgen, Novo Nordisk, Optoceutics, Passage Bio, Pinteon Therapeutics, Prothena, Red Abbey Labs, reMYND, Roche, Samumed, Siemens Healthineers, Triplet Therapeutics, and Wave; has given lectures in symposia sponsored by Cellectricon, Fujirebio, Alzecure, Biogen, and Roche; and is a co‐founder of Brain Biomarker Solutions in Gothenburg AB (BBS), which is a part of the GU Ventures Incubator Program (outside submitted work). D.A.G. has received honorarium for speaking from Ipsen, and honorarium for consulting from Allergan and Abbvie. M.F. is listed on a patent related to methods and kits for differential diagnosis of Alzheimer disease versus frontotemporal dementia using blood biomarkers. M.B. received royalties/licenses from Roche, and consulting fees from Biogen. RHS reports ownership in FollowMD Inc., a vascular risk reduction clinic. T.K.R. participated in 2021 and 2022 in an advisory activity for Biogen Canada Inc. T.K.R. is also an inventor on the United States Provisional Patent No. 17/396,030 that describes cell‐based assays and kits for assessing serum cholinergic receptor activity. Unrelated to this work, M.M. reports personal fees from Henry Stewart Talks, Alector, Biogen Canada, and Wave Life Sciences; as well as grants from Roche, Alector and Washington University. E.S., T.W., G.C., S.M., A.A.B., J.R., M.A.B., S.E.B., A.A.D., R.A.D., D.D., S.F., E.F., C.E.F., A.F., R.A.G., A.H., R.A.H., S.K., A.E.L., C.M., P.M.M., J.B.O., S.H.P., B.G.P., A.C.R., J.F.R., E.R., D.J.S., G.S., M.J.S., D.F.T., M.C.T., A.K.T., H.K., and D.P.M. report no conflicts. Author disclosures are available in the supporting information.

## CONSENT STATEMENT

Research ethics committees at all participating recruitment sites approved the ONDRI research protocol. All participants and study partners provided written and informed consent prior to participation and in accordance with the Declaration of Helsinki.

## Supporting information

Supporting Information

Supporting Information

## Data Availability

Curated data from the ONDRI cohort are available through controlled release on Brain‐CODE (www.braincode.ca). The list of ONDRI datasets used in this study can be found in Table S3 in supporting information.

## COLLABORATORS—THE ONDRI INVESTIGATORS

The current list can be found at https://ondri.ca/team/ondri‐researchers. The list below (last name, first name) is as of June 20, 2023.

**Table:**

Abrahao, Agessandro	Arnott, Stephen R.	Bartha, Robert
Beaton, Derek	Berberian, Stephanie	Breen, David E.
Bronskill, Susan E.	Bulman, Dennis E.	Casaubon, Leanne K.
Chen, Ying	Cheng, Richard W.	Coe, Brian C.
Cornish, Benjamin	Datye, Asim	Edwards, Jodi D.
Fan, Carina L.	Favaro Polastri, Paula	Fishman, Keera N.
Fraser, Julia E.	Godkin, F. Elizabeth	Gutierrez, Stephanie
Haddad, Seyyed M.H.	Harris, Daniel A.	Hatch, Wendy V.
Havens, Alexander	Huang, Jeff	Hudson, Christopher
Iaboni, Andrea	Jaakkimainen, Liisa	Jog, Mandar S.
Jung, Hyejung	Kapral, Moira K.	Khoo, Ben
Kwan, Donna	Latapi, Paulina	Lawrence‐Dewar, Jane
Levine, Brian	Lim, Andrew S. P.	Lou, W. Y. Wendy
Lyra, Natalia	Maclagan, Laura C.	Malik, Rubina
Mandzia, Jennifer	Maxwell, Colleen J.	McCreath, Graham
McIlroy, W.E. Bill	Meltzer, Jed	Montero‐Odasso, Manuel
Munoz, David	Nanayakkara, Nuwan D.	Ozzoude, Miracle
Parokaran Varghese, Jessy	Pieruccini‐Faria, Frederico	Riek, Heidi
Richter, Richard	Robertson, Andrew D.	Robinson, John D.
Santos, Haylie	Scott, Christopher J.M.	Seitz, Dallas
Shaikh, Komal	Shoesmith, Christen	Southwell, Alisia
Steeves, Thomas	Strother, Stephen C.	Sulistyo, Adrienne
Sunderland, Kelly M.	Swardfager, Walter	Symons, Sean
Tan, Brian	Tatham, Erica L.	Tayyari, Faryan
Thomson, Sherri Lynn	Turnbull, John	Van Ooteghem, Karen
Venkatesh, Raadhika	Weber, Kyle	Wong, Bryan
Yhap, Vanessa	Yu, Amy	Yu, Di
Zaidi, Khush‐Bakht	Zarei, Shadi	Zhu, Julie
Zinman, Lorne	Zou, Guangyong	
