# Supplementary material for: Association of plasma biomarkers with cognition, cognitive decline, and daily function across and within neurodegenerative diseases: Results from the Ontario Neurodegenerative Disease Research Initiative
Source: Alzheimers Dement. 2023 Dec 17;20(3):1753–70. doi: 10.1002/alz.13560 (PMC10984487; doi:10.1002/alz.13560)
Supplement: Supplementary file 1 — Supporting Information [file ALZ-20-1753-s001.docx]

**Supplementary material**

**1. Derivation of cognitive domain composite scores**

Composite scores were derived from 26 raw test scores for the following five cognitive domains: attention & working memory, executive function, language, memory, visuospatial function. The full list of neuropsychological tests by cognitive domain is presented in Supplementary Table S1. First, a series of linear regressions were fitted with each raw score as the dependent variable, and age, sex, and years of education as independent variables. The residuals of these linear regressions, representing the raw scores with the observed effects of age, sex, and education removed, were used to calculate the standard deviations across all participants at baseline. Residuals from both baseline and follow-up visits were divided by these standard deviations to obtain z-scores. For timed tests, z-scores were inverted to ensure that higher scores are consistent with better performance. Finally, z-scores were averaged across tests to generate the five cognitive domain composite scores according to Supplementary Table S1.

Regarding missing raw data, in the few specific cases of extensive cognitive impairment precluding test completion, the lowest possible or chance equivalent raw score was inputted as it is assumed to be the score the participant would have obtained. Supplementary Table S2 details the number of imputations per test. Missing data due to other reasons were excluded. A two-test threshold was then used for composite score calculations; composite scores were not calculated for cognitive domains with less than two valid tests remaining. Almost no participants were excluded per cognitive domain for missing data at baseline (FTD n=1), and very few longitudinally (AD/MCI n=2 to 6, PD n=3 to 5, FTD n=2 to 7, CVD n=1 to 4).

**2. Additional analyses with plasma Aβ_42_**

All analyses presented in the main paper were repeated here with plasma Aβ_42_ (log), with imputed values for samples below the functional limit of detection (1.51 pg/mL, imputation=limit/2). As Aβ_42/40_ ratios were not calculated when concentration of either Aβ_42_ or Aβ_40_ were below the functional limit of quantification, these supplementary analyses allow us to explore if there could be valuable information in those missing ratios. Similarly to the main Aβ_42/40_ ratio results, almost no associations were found, supporting the idea that amyloid-β markers are not the most effective in already diagnosed neurodegenerative populations. No significant associations were found in linear mixed models in the pooled diseases group, in the ADMCI group, in the PD group, and in the CVD group, for baseline or longitudinal results. In the FTD group, lower Aβ_42_ levels were significantly associated with worse visuospatial function scores at baseline (B=1.78, *P*=0.026), and with less longitudinal decline in attention & working memory (B=-0.067, *P*=0.029) and in executive function (B=-0.46, *P*=0.028).

**3. Associations between plasma biomarkers and cognition within the healthy control group**

The association between baseline plasma biomarkers (GFAP, NfL, p-tau181, Aβ_42/40_) and baseline cognitive domains (attention & working memory, executive function, language, memory, visuospatial function) was assessed using linear regression models, within the healthy control group. *APOE* E4 carrier status was included as covariate. Age, sex, and education were accounted for during initial computing of cognitive composite scores. No significant associations were found.

**4. Plasma biomarkers inter-correlations**

Spearman correlations were performed to explore inter-correlations between plasma biomarkers. As expected, all plasma biomarkers showed some level of association: GFAP, NfL, and p-tau181 levels were all positively associated with each other, and negatively associated with Aβ_42/40_ ratio. The correlation matrix is presented in Supplementary table S3.

**5. Additional analyses with MoCA**

The association between baseline plasma biomarkers (GFAP, NfL, p-tau181, Aβ_42/40_) and baseline MoCA (Montreal Cognitive Assessment) total scores was assessed using linear regression models, across the ONDRI cohort. Age, sex, education, and *APOE* E4 carrier status were included as covariates. Results were similar to what was found with individual composite cognitive domains. Higher levels of GFAP, NfL, and p-tau181 were significantly associated with worse MoCA total scores (β=-0.25, *P*<0.001; β=-0.19, *P*<0.001; β=-0.22, *P*<0.001; respectively). Lower Aβ_42/40_ ratio was trending towards a similar association with worse MoCA total scores (β=0.09, P=0.053).

**6. Effect of kidney and liver function on plasma biomarker levels and their association with cognitive function**

The association between markers of kidney and liver function and plasma biomarker levels is detailed in Supplementary Table S5. Markers of liver function (aspartate transaminase and alanine transaminase) were not significantly associated with any plasma biomarkers. Kidney function (estimated glomerular filtration rate, CKD-EPI equation) was significantly associated with GFAP, NfL, and p-tau181. Higher glomerular filtration rate was associated with lower levels of all three plasma biomarkers. Supplementary Table S6 details the association between baseline plasma biomarkers and cognitive function, both baseline and longitudinal rate of change, when glomerular filtration rate is accounted for in addition to previous covariates. The addition of glomerular filtration rate as a covariate to the models did not affect any results: all results are either identical or very slightly more significant.

**Supplementary Tables**

**Supplementary Table S1. Full list of neuropsychological tests by cognitive domain**

| **Cognitive Domain** | **Individual tests by domain** |
| --- | --- |
| Attention & working memory | Symbol Digit Modality Test (coding)  Trail Making Test – Part A (time)  WAIS-III: Digit Span Forward  WAIS-III: Digit Span Backward  WAIS-III: Digit Span Total  DKEFS: Color Naming (time)  DKEFS: Word Reading (time) |
| Executive function | Trail Making Test – Part B (time)  DKEFS: Interference (time)  DKEFS: Switching (time)  DKEFS: Letter Fluency  DKEFS: Category Fluency  WAIS-II: Matrix Reasoning |
| Language | Boston Naming – 15 Item (pro-rated)  TAWF: Verb Naming  BDAE: Semantic Probe  WAIS-II: Vocabulary |
| Memory | RAVLT: Immediate  RAVLT: Long-delay  RAVLT: Recognition Discrimination  BVMT-R: Immediate  BVMT-R: Delayed  BVMT-R: Recognition Discrimination |
| Visuospatial function | Judgement of Line Orientation  VOSP: Incomplete Letters  BVMT-R: Copy Trial |

**Supplementary Table S2. Number of imputations per neuropsychological test**

| **Neuropsychological test** | **Number of imputations** | | |
| --- | --- | --- | --- |
|  | **Baseline** | **One-year follow-up** | **Two-year follow-up** |
| Symbol Digit Modality Test | 1 | 1 | 1 |
| Trail Making Test – Part A | 0 | 3 | 1 |
| WAIS-III: Digit Span Forward | 0 | 2 | 0 |
| WAIS-III: Digit Span Backward | 0 | 2 | 2 |
| WAIS-III: Digit Span Total | 0 | 2 | 2 |
| DKEFS: Color Naming | 1 | 3 | 1 |
| DKEFS: Word Reading | 1 | 2 | 1 |
| Trail Making Test – Part B | 1 | 11 | 10 |
| DKEFS: Interference | 4 | 15 | 13 |
| DKEFS: Switching | 25 | 35 | 33 |
| DKEFS: Letter Fluency | 0 | 0 | 1 |
| DKEFS: Category Fluency | 0 | 0 | 1 |
| WAIS-II: Matrix Reasoning | 0 | 1 | 1 |
| Boston Naming – 15 Item | 0 | 0 | 1 |
| TAWF: Verb Naming | 0 | 0 | 2 |
| BDAE: Semantic Probe | 1 | 3 | 5 |
| WAIS-II: Vocabulary | 0 | 1 | 1 |
| RAVLT: Immediate | 1 | 2 | 3 |
| RAVLT: Long-delay | 1 | 2 | 4 |
| RAVLT: Recognition Discrimination | 1 | 2 | 4 |
| BVMT-R: Immediate | 0 | 1 | 0 |
| BVMT-R: Delayed | 0 | 1 | 0 |
| BVMT-R: Recognition Discrimination | 0 | 1 | 0 |
| Judgement of Line Orientation | 7 | 10 | 7 |
| VOSP: Incomplete Letters | 0 | 0 | 0 |
| BVMT-R: Copy Trial | 0 | 1 | 0 |

**Supplementary Table S3. ONDRI datasets**

| **Category** | **Name of dataset** |
| --- | --- |
| ***Plasma biomarkers*** |  |
| AD/MCI | OND01_ADMCI_01_GNMC_PROTEIN_2022JUL19_DATA |
| PD | OND01_PD_01_GNMC_PROTEIN_2022JUL19_DATA |
| FTD | OND01_FTD_01_GNMC_PROTEIN_2022JUL19_DATA |
| CVD | OND01_VCI_01_GNMC_PROTEIN_2022JUL19_DATA |
| ***Genotyping*** |  |
| AD/MCI | OND01_ADMCI_01_GNMC_CANPATH_2018AUG07_DATA |
| PD | OND01_PD_01_GNMC_CANPATH_2018AUG07_DATA |
| FTD | OND01_FTD_01_GNMC_CANPATH_2018AUG07_DATA |
| CVD | OND01_VCI_01_GNMC_CANPATH_2018AUG07_DATA |
| ***Neuropsychology*** |  |
| AD/MCI | OND01_ADMCI_01_NPSY_FULL_2020DEC16_DATA |
| AD/MCI | OND01_ADMCI_04_NPSY_FULL_2021APR19_DATA |
| AD/MCI | OND01_ADMCI_06_NPSY_FULL_2022MAY02_DATA |
| PD | OND01_PD_01_NPSY_FULL_2020DEC16_DATA |
| PD | OND01_PD_04_NPSY_FULL_2020DEC14_DATA |
| PD | OND01_PD_06_NPSY_FULL_2022APR29_DATA |
| FTD | OND01_FTD_01_NPSY_FULL_2020DEC16_DATA |
| FTD | OND01_FTD_04_NPSY_FULL_2021APR19_DATA |
| FTD | OND01_FTD_06_NPSY_FULL_2022FEB17_DATA |
| CVD | OND01_VCI_01_NPSY_FULL_2020DEC16_DATA |
| CVD | OND01_VCI_04_NPSY_FULL_2021APR13_DATA |
| CVD | OND01_VCI_06_NPSY_FULL_2023MAR03_DATA |

**Supplementary Table S4. Spearman correlation matrix for plasma biomarkers**

|  | **GFAP Log(pg/mL)** | **NfL Log(pg/mL)** | **P-tau181 Log(pg/mL)** | **Aβ_42/40_ Log(ratio)** |
| --- | --- | --- | --- | --- |
| **GFAP Log(pg/mL)** |  | r=0.53  *P*<0.001 | r=0.34  *P*<0.001 | r=-0.21  *P*<0.001 |
| **NfL Log(pg/mL)** |  |  | r=0.35  *P*<0.001 | r=-0.14  *P*=0.003 |
| **P-tau181 Log(pg/mL)** |  |  |  | r=-0.14  *P*=0.002 |
| **Aβ_42/40_ Log(ratio)** |  |  |  |  |

**Supplementary Table S5. Spearman correlations between glomerular filtration rate, aspartate transaminase, alanine transaminase and plasma biomarkers**

|  | **Glomerular filtration rate** | **Aspartate transaminase** | **Alanine transaminase** |
| --- | --- | --- | --- |
| **GFAP Log(pg/mL)** | r=-0.29  *P*<0.001 | ns | ns |
| **NfL Log(pg/mL)** | r=-0.35  *P*<0.001 | ns | ns |
| **P-tau181 Log(pg/mL)** | r=-0.15  *P*=0.001 | ns | ns |
| **Aβ_42/40_ Log(ratio)** | ns | ns | ns |

**Supplementary Table S6. Association between plasma biomarkers and cognitive function in the ONDRI cohort, additionally controlling for glomerular filtration rate**

|  |  | **GFAP** | **NfL** | **P-tau181** | **Aβ_42/40_** |
| --- | --- | --- | --- | --- | --- |
| **Baseline** | **Attention & working memory** | B=-0.57  *P*<0.001 | B=-0.67  *P*<0.001 | ns | ns |
|  | **Executive function** | B=-0.70  *P*<0.001 | B=-0.77  *P*<0.001 | B=-0.26  *P*=0.034 | ns |
|  | **Language** | B=-0.38  *P*=0.032 | B=-0.63  *P*=0.001 | ns | ns |
|  | **Memory** | B=-0.66  *P*<0.001 | B=-0.46  *P*=0.005 | B=-0.34  *P*=0.005 | ns |
|  | **Visuospatial function** | B=-0.36  *P*=0.019 | ns | ns | ns |
| **Longitudinal rate of change** | **Attention & working memory** | B=-0.014  *P*<0.001 | B=-0.014  *P*=0.002 | B=-0.008  *P*=0.016 | ns |
|  | **Executive function** | B=-0.013  *P*<0.001 | B=-0.011  *P*=0.004 | ns | ns |
|  | **Language** | B=-0.018  *P*=0.002 | B=-0.023  *P*<0.001 | ns | ns |
|  | **Memory** | ns | B=-0.011  *P*=0.016 | ns | ns |
|  | **Visuospatial function** | B=-0.020  *P*=0.001 | B=-0.018  *P*=0.010 | ns | ns |
